# Autophagy induction by SIRT6 is involved in oxidative stress-induced neuronal damage

**DOI:** 10.1007/s13238-016-0257-6

**Published:** 2016-03-16

**Authors:** Jiaxiang Shao, Xiao Yang, Tengyuan Liu, Tingting Zhang, Qian Reuben Xie, Weiliang Xia

**Affiliations:** State Key Laboratory of Oncogenes and Related Genes, Renji-Med X Stem Cell Research Center, Ren Ji Hospital, School of Biomedical Engineering, Shanghai Jiao Tong University, Shanghai, 200030 China

**Keywords:** SIRT6, oxidative stress, neuronal damage, autophagy, AKT

## Abstract

SIRT6 is a NAD^+^-dependent histone deacetylase and has been implicated in the regulation of genomic stability, DNA repair, metabolic homeostasis and several diseases. The effect of SIRT6 in cerebral ischemia and oxygen/glucose deprivation (OGD) has been reported, however the role of SIRT6 in oxidative stress damage remains unclear. Here we used SH-SY5Y neuronal cells and found that overexpression of SIRT6 led to decreased cell viability and increased necrotic cell death and reactive oxygen species (ROS) production under oxidative stress. Mechanistic study revealed that SIRT6 induced autophagy via attenuation of AKT signaling and treatment with autophagy inhibitor 3-MA or knockdown of autophagy-related protein Atg5 rescued H_2_O_2_-induced neuronal injury. Conversely, SIRT6 inhibition suppressed autophagy and reduced oxidative stress-induced neuronal damage. These results suggest that SIRT6 might be a potential therapeutic target for neuroprotection.

**Electronic supplementary material:**

The online version of this article (doi:10.1007/s13238-016-0257-6) contains supplementary material, which is available to authorized users.

## INTRODUCTION

Sirtuins are a family of NAD^+^-dependent histone deacetylases involved in diverse cellular functions including inflammation, energy metabolism, stress resistance and cancer (Haigis and Sinclair, 2010). There are seven mammalian sirtuins with varied subcellular localizations and enzymatic activities (Michishita et al., 2005). SIRT6 is predominantly localized in the nucleus, functioning as an ADP-ribosyltransferase and NAD^+^-dependent deacetylase (Liszt et al., 2005; Michishita et al., 2008; Feldman et al., 2013). SIRT6 plays an important role in genomic stability, DNA repair, metabolic homeostasis and diseases such as obesity, cardiac hypertrophy and cancer (Liu et al., 2013; Kugel and Mostoslavsky, 2014). A recent study has reported that SIRT6 is associated with high mobility group box-1 (HMGB1) release after cerebral ischemia, but knockdown of SIRT6 has no effect on neuronal cell death induced by oxygen and glucose deprivation (OGD) (Lee et al., 2013b). Since oxidative stress is a key mediator of tissue damage after ischemia-reperfusion, the role of SIRT6 in oxidative stress-induced neuronal injury remains to be investigated.

Autophagy is a catabolic process which involves degradation of unnecessary cellular components or dysfunctional organelles in response to metabolic demand and diverse stress (Kroemer et al., 2010). Whether the autophagic process is beneficial or detrimental remains controversial. The term ‘autophagic cell death’ has been introduced to describe cell death that can be suppressed by inhibition of the autophagy (Galluzzi et al., 2012). Autophagy is regulated by diverse signaling pathways and stressful conditions including nutrient deprivation, hypoxia and oxidative stress (He and Klionsky, 2009). Reactive oxygen species (ROS) serve as triggers of autophagy since antioxidants treatment could to some extent rescue the autophagic programme (Filomeni et al., 2014). In this study, we aimed to uncover the role of SIRT6 in oxidative stress-induced cell death and the possible mechanisms like autophagy.

## RESULTS

### Oxidative stress causes an acute reduction in SIRT6 expression

**Figure 1:**
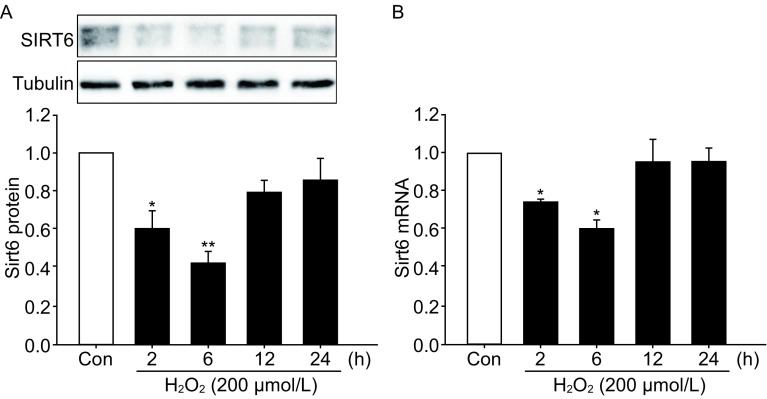
SIRT6 expression after H_2_O_2_ treatment. SH-SY5Y cells were exposed to 200 µmol/L H_2_O_2_ for 1 h and then cultured for indicated time. SIRT6 protein (A) and mRNA (B) levels were detected by Western blot and qPCR. Bars represent the mean ± SEM from three independent experiments. *P < 0.05; **P < 0.01, compared with the control group

SH-SY5Y cells were exposed to H_2_O_2_ for 1 h and then cultured for indicated time. SIRT6 protein levels were decreased at 2 h and 6 h, but recovered to almost basal levels at 12 h and 24 h post H_2_O_2_ treatment (Fig. 1A). Similar change pattern was also observed in SIRT6 mRNA levels (Fig. 1B).

### SIRT6 enhances oxidative stress-induced neuronal cell death

**Figure 2:**
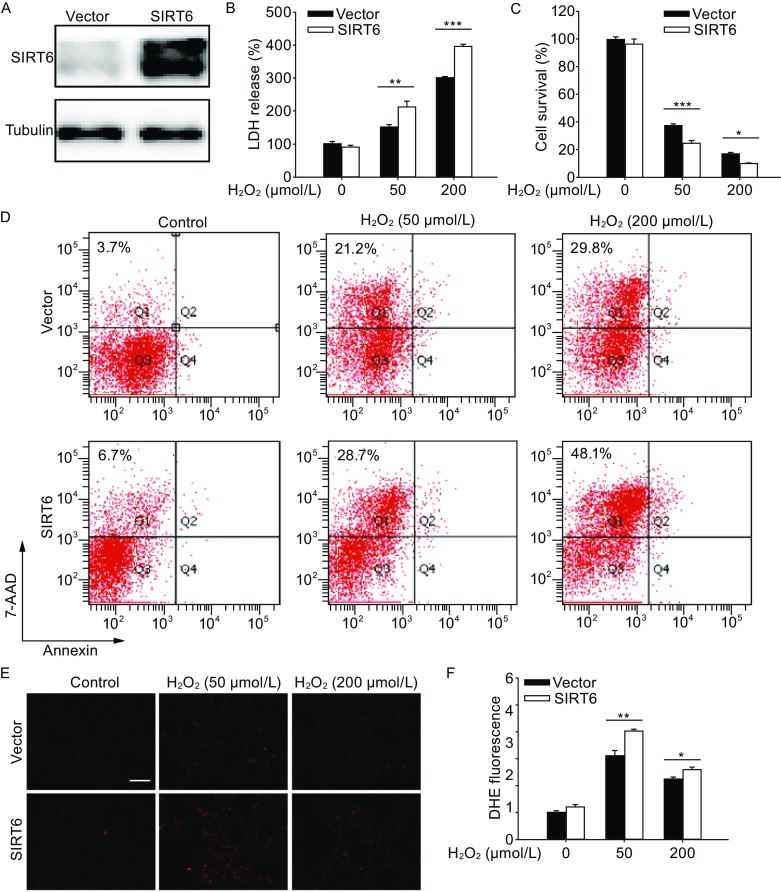
SIRT6 enhances H_2_O_2_-induced cell death and ROS accumulation. (A) Western blot analysis of SH-SY5Y cells stably transfected with vector or SIRT6 lentivirus. (B and C) SH-SY5Y cells were pretreated with H_2_O_2_ for 1 h and then cultured for 24 h. Cell death was assessed by LDH release assay and cell viability was assessed by CCK-8 assay. (D) Necrosis and apoptosis were assessed by Annexin V/7-AAD assay. (E and F) SH-SY5Y cells were pretreated with H_2_O_2_ for 1 h and then cultured for 6 h. Intracellular ROS levels were visualized by DHE staining and quantified. Bars represent the mean ± SEM from at least three independent experiments. Scale bar = 100 µm, *P < 0.05; **P < 0.01; ***P < 0.001

To investigate the potential effect of SIRT6 on oxidative stress-induced neuronal injury, SH-SY5Y cells stably infected with vector or SIRT6 lentivirus (Fig. 2A) were exposed to H_2_O_2_ for 1 h and then cultured for 24 h. We found that SIRT6-overexpressing cells showed significantly higher death levels and lower viability levels than their counterparts (Fig. 2B and 2C). Similar results were also observed in PC12 cells transfected with vector or SIRT6 plasmids (Fig. S1A and S1B). Flow cytometry analysis also revealed that H_2_O_2_ treatment caused a remarkable increase in cell necrosis (Annexin V^−^/7-AAD^+^) and overexpression of SIRT6 raised the proportion of necrotic cells (Fig. 2D). Moreover, SIRT6 overexpression upregulated the intracellular ROS production as shown by the DHE staining (Fig. 2E and 2F).

### SIRT6 induces autophagy under oxidative stress

**Figure 3:**
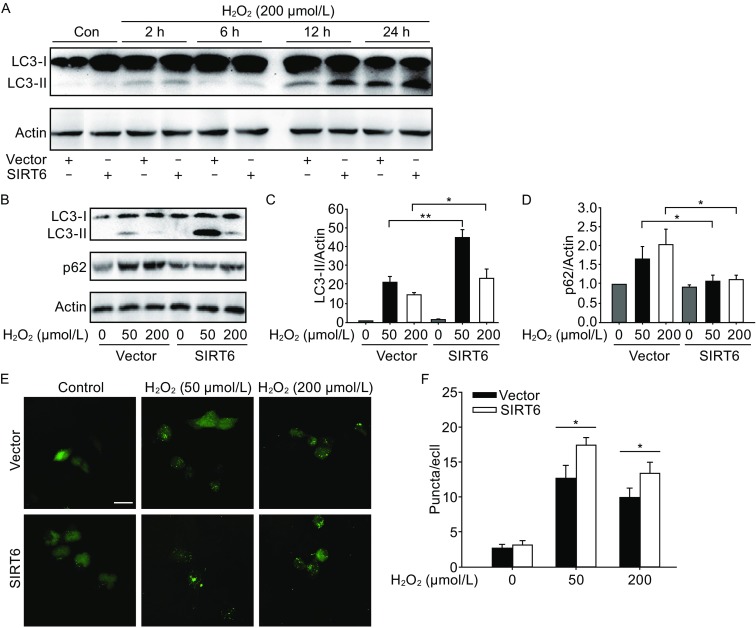
SIRT6 induces autophagy after H_2_O_2_ treatment. (A) SH-SY5Y cells were pretreated with H_2_O_2_ for 1 h and then cultured for indicated time. Cell lysates were analyzed by immunoblotting with antibodies specific to LC3. (B–D) SH-SY5Y cells were pretreated with H_2_O_2_ for 1 h and then cultured for 24 h. LC3 and p62 levels were analyzed by Western blot and quantified. (E and F) SH-SY5Y cells were exposed to H_2_O_2_ for 1 h and then cultured for 24 h after transfection with EGFP-LC3 plasmid. Number of EGFP-LC3 puncta per cell was counted to quantify autophagosome formation. Bars represent the mean ± SEM from at least three independent experiments. Scale bar = 20 µm, *P < 0.05; **P < 0.01

**Figure 4:**
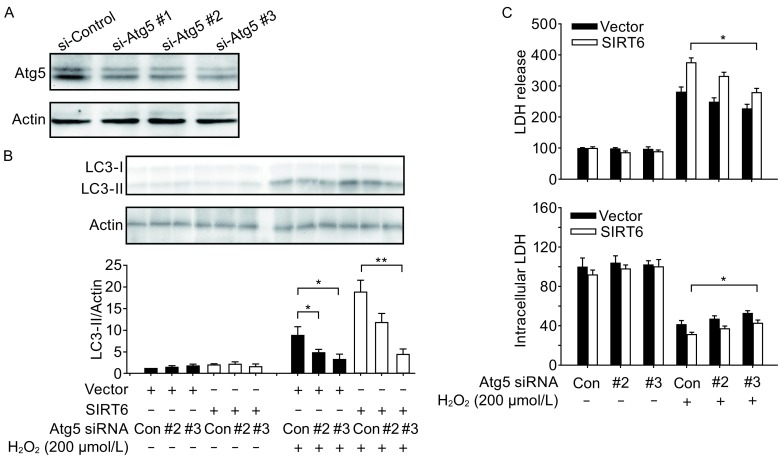
SIRT6-regulated autophagy is responsible for H_2_O_2_-induced neuronal cell death. (A) Knockdown of Atg5 by siRNAs. (B) SH-SY5Y cells were exposed to H_2_O_2_ for 1 h and then cultured for 24 h after transfection with Atg5 siRNAs. LC3 levels were analyzed by Western blot and quantified. (C) SH-SY5Y cells were exposed to H_2_O_2_ for 1 h and then cultured for 24 h after transfection with Atg5 siRNAs. Cell death and viability were assessed by LDH assay. Bars represent the mean ± SEM from at least three independent experiments. *P < 0.05; **P < 0.01

A previous study has reported that SIRT6 modulates human bronchial epithelial cell (HBEC) senescence via induction of autophagy (Takasaka et al., 2014), therefore we investigated whether SIRT6 could regulate autophagy in the neuronal cells. Exposure to H_2_O_2_ upregulated the levels of autophagy marker LC3-II in a time-course manner (Fig. 3A). SIRT6 induced autophagy as shown by increased conversion to LC3-II and decreased accumulation of p62 in SH-SY5Y cells as well as in PC12 cells (Figs. 3B–D, S1C and S1D). Formation of autophagosome puncta containing LC3 has been regarded as a hallmark of autophagic activation. We transfected SH-SY5Y cells with EGFP-LC3 plasmid and detected the autophagic puncta after H_2_O_2_ treatment. There were more EGFP-LC3 puncta in SIRT6 group than in vector group (Fig. 3E and 3F). To assess the role of autophagy in oxidative stress-induced neuronal damage, we treated cells with a specific autophagy inhibitor 3-MA, which could reduce SIRT6-mediated neuronal cell death (Fig. S2A and S2B). Furthermore, we downregulated the expression of Atg5, an E3 ubiquitin ligase necessary for autophagy, in SH-SY5Y cells (Fig. 4A). Knockdown of Atg5 inhibited SIRT6-mediated autophagy (Fig. 4B) and diminished the susceptibility to oxidative stress in SIRT6-overexpressing cells (Fig. 4C). Taken together, these data suggest that autophagy is responsible for oxidative stress-induced neuronal injury.

### SIRT6 attenuates AKT signaling under oxidative stress

**Figure 5:**
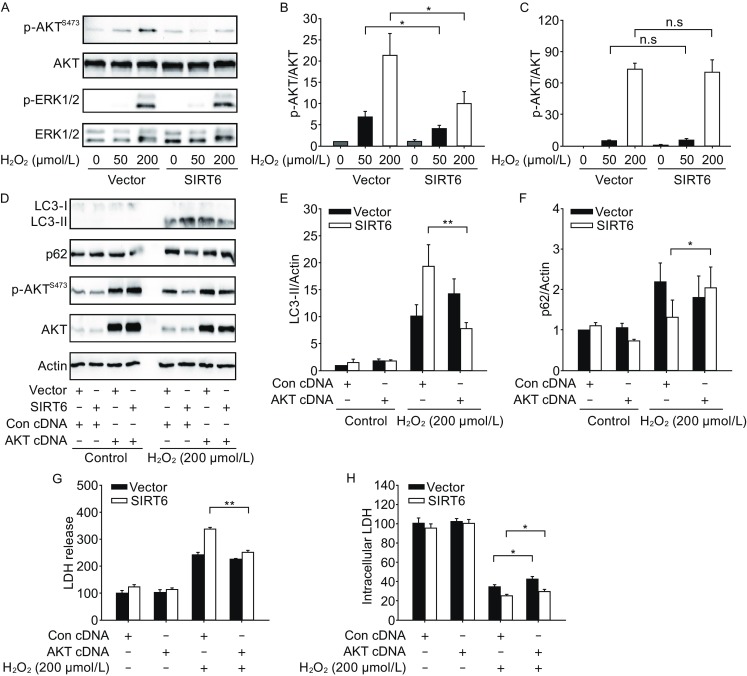
SIRT6 attenuates AKT signaling after H_2_O_2_ treatment. (A–C) SH-SY5Y cells were pretreated with H_2_O_2_ for 1 h and then cultured for 24 h. Phosphorylated AKT and ERK were analyzed by Western blot and quantified. (D–F) SH-SY5Y cells were exposed to H_2_O_2_ for 1 h and then cultured for 24 h after transfection with MYR-AKT plasmid. LC3 and p62 levels were analyzed by Western blot and quantified. (G and H) SH-SY5Y cells were exposed to H_2_O_2_ for 1 h and then cultured for 24 h after transfection with MYR-AKT plasmid. Cell death and viability were assessed by LDH assay. Bars represent the mean ± SEM from at least three independent experiments. *P < 0.05; **P < 0.01

Next we asked whether SIRT6 could modulate autophagy-related signaling cascades. AKT/mTOR and MAPK pathways are the main upstream regulators of autophagy (He and Klionsky, 2009). H_2_O_2_ treatment caused AKT and ERK MAPK activation in a time-course manner (Fig. S3). SIRT6 suppressed phosphorylation of AKT in SH-SY5Y cells as well as in PC12 cells, but phosphorylation of ERK MAPK was not affected (Figs. 5A–C, S1C and S1D). To validate the role of AKT pathway in SIRT6 function, we performed rescue experiments by introducing a constitutively active myristoylated form of AKT into SH-SY5Y cells (Domingo-Domenech et al., 2012). Forced expression of constitutively active AKT ameliorated SIRT6-mediated autophagy induction (Fig. 5D–F) and reduced neuronal cell death (Fig. 5G and 5H), indicating that AKT pathway is involved in oxidative stress damage. Similar results were also observed in cells exposed to lower concentration (50 µmol/L) of H_2_O_2_ (Fig. S4A and S4B).

### SIRT6 inhibition reduces neuronal cell death and attenuates autophagy under oxidative stress

**Figure 6:**
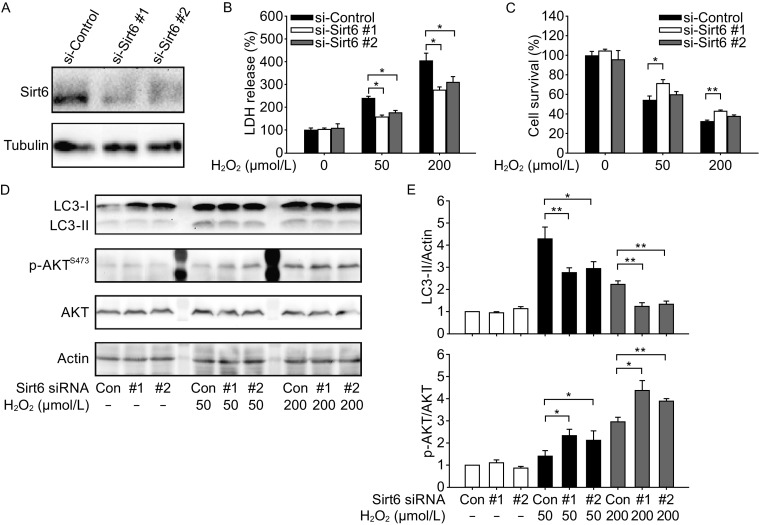
SIRT6 inhibition reduces neuronal cell death and attenuates autophagy after H_2_O_2_ treatment. (A) Knockdown of Sirt6 by siRNAs. (B and C) SH-SY5Y cells were exposed to H_2_O_2_ for 1 h and then cultured for 24 h after transfection with Sirt6 siRNAs. Cell death was assessed by LDH release assay and cell viability was assessed by CCK-8 assay. (D and E) SH-SY5Y cells were exposed to H_2_O_2_ for 1 h and then cultured for 24 h after transfection with Sirt6 siRNAs. LC3 and phosphorylated AKT levels were analyzed by Western blot and quantified. Bars represent the mean ± SEM from at least three independent experiments. *P < 0.05; **P < 0.01

Besides the gain-of-function study, we used siRNAs to knock down SIRT6 in SH-SY5Y cells (Fig. 6A). SIRT6 inhibition decreased cell death and increased cell survival after exposure to H_2_O_2_ (Fig. 6B and 6C). Furthermore, SIRT6 inhibition attenuated autophagy and activated AKT signaling pathway (Fig. 6D and 6E). Thus, these data were complementary with those from SIRT6 overexpression experiments, confirming the role of SIRT6 in oxidative stress-induced neuronal damage.

## DISCUSSION

**Figure 7:**
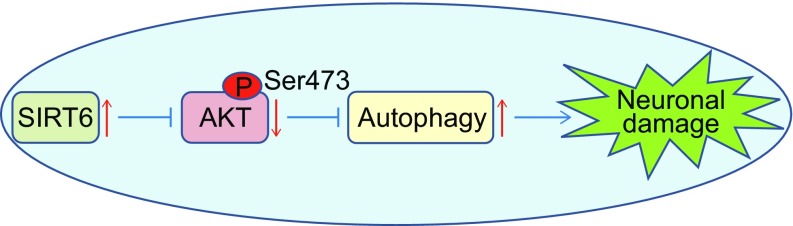
A schematic to summarize the role of SIRT6 in oxidative stress-induced neuronal damage. SIRT6 enhances neuronal cell death under oxidative stress via induction of autophagy mediated by attenuation of AKT signaling

In this study, we report that SIRT6 enhances oxidative stress-induced damage in neuronal cells, which is associated with increased necrotic cell death and ROS production. Mechanistic study reveals that SIRT6 induces autophagy via attenuation of AKT signaling. Furthermore, pharmacological or genetic inhibition of autophagy and forced expression of constitutively active AKT could rescue SIRT6-mediated neuronal damage (Fig. 7).

Previous studies have reported that SIRT6 levels are downregulated under stressful conditons such as cellular senescence or oxygen and glucose deprivation (Lee et al., 2013b; Liu et al., 2014; Takasaka et al., 2014). Similar to the above results, we found that SIRT6 levels showed an acute decrease following H_2_O_2_ treatment, but restored to almost basal levels during longer time. This might be explained by the reason that we detected SIRT6 levels upon a transient stimuli, since cells were replaced with fresh medium and cultured for different time after exposure to H_2_O_2_. The acute decrease in both protein and mRNA levels indicates that SIRT6 expression might be regulated by proteasomal degradation or micro-RNAs. Ubiquitin ligase CHIP could prevent degradation of SIRT6 through noncanonically ubiquitinating SIRT6 at K170 (Ronnebaum et al., 2013). CHIP protein is reduced under oxidative stress (Lee et al., 2013a), thus it is possible that inhibition of SIRT6 degradation by CHIP will be removed after H_2_O_2_ treatment. Another ubiquitin-specific peptidase USP10 could also deubiquitinate SIRT6 protecting it from proteasomal degradation (Lin et al., 2013). Activation of AKT1 phosphorylates SIRT6 leading to interaction and ubiquitination of SIRT6 by MDM2 (Thirumurthi et al., 2014), thus oxidative stress-induced AKT activation will cause ubiquitination-mediated degradation of SIRT6. Furthermore, micro-RNAs targeting SIRT6 have also been reported, including miR-33b, miR-34a and miR-766 (Davalos et al., 2011; Lefort et al., 2013; Sharma et al., 2013). Ischemia/Reperfusion (IR) induces miR-34a expression in the rat hepatocytes (Huang et al., 2014), which makes the possibility that upregulation of miR-34a under oxidative stress might lead to decrease in SIRT6 levels.

Oxidative stress induces autophagy in different type of cells such as cardiomyocytes, auditory cells, endothelial progenitor cells and embryonic stem cells (Dutta et al., 2013; Ou et al., 2014; Hayashi et al., 2015; Tang et al., 2015). Here we confirmed that oxidative stress could induce autophagy in neuronal cells, as H_2_O_2_ treatment upregulated LC3-II levels in a time course-dependent manner. The role of autophagy in the oxidative stress damage remains controversial. Curcumin protects vascular endothelial cell from H_2_O_2_-induced cytotoxicity through induction of autophagy (Han et al., 2012), whereas adiponectin inhibits H_2_O_2_-mediated autophagosome formation and prevents loss of cell viability in cardiomyocytes (Essick et al., 2013). In this study, we showed that SIRT6-mediated autophagy contributes to oxidative stress-induced neuronal injury, since inhibition of autophagy by specific inhibitor 3-MA or knockdown of Atg5 could rescue the detrimental effect of SIRT6 on cell survival. Recently it has been reported that rAAV-mediated SIRT6 overexpression downregulated cell viability in primary neurons under oxidative stress (Cardinale et al., 2014), which agrees with our present results.

The mammalian target of rapamycin (mTOR) is a crucial negative regulator of autophagy and AKT is an upstream signaling molecule for mTOR activation (Arico et al., 2001; Manning et al., 2002). Previous studies have shown the corelation between SIRT6 and AKT signaling. SIRT6 extends lifespan in male mice or prevents cardiac hypertrophy via the attenuation of IGF-AKT pathways (Kanfi et al., 2012; Sundaresan et al., 2012). Recently it has been reported that autophagy induction by SIRT6 through attenuation of insulin-like growth factor signaling is involved in human bronchial epithelial cell senescence (Takasaka et al., 2014). In this study, SIRT6 enhances oxidative stress-induced autophagy associated with decreased phosphorylation of AKT, which is in line with the above observations. Moreover, activation of AKT1 phosphorylates and promotes the degradation of SIRT6 (Thirumurthi et al., 2014), thus there may exist a positive-feedback loop between SIRT6 and AKT. Ras-Raf1-MEK cascade is another signaling pathway regulating autophagy (Pattingre et al., 2003; Furuta et al., 2004). We showed that oxidative stress causes a dramatic increase in phosphorylation of ERK1/2, but SIRT6 overexpression does not affect ERK1/2 signaling pathway.

In conclusion, we demonstrated that autophagy induction by SIRT6 is involved in oxidative stress-induced neuronal cell death, which could be attributed to attenuation of AKT signaling. Therefore, regulation of autophagy by controlling SIRT6 levels might be a promising therapeutic target for ischemia and other brain diseases.

## MATERIALS AND METHODS

### Reagents

Hydrogen peroxide (H_2_O_2_) (323381) and 3-methyladenine (3-MA) (M9281) were purchased from Sigma Aldrich (St. Louis, MO). MYR-AKT (#9005) and EGFP-LC3 (#21073) plasmids were from Addgene. Atg5 and Sirt6 siRNAs were from GenePharma (Shanghai, China).

### Cell culture

SH-SY5Y human neuroblastoma cells were from Institute of Neurology, Ruijin Hospital (Shanghai, China). Differentiated PC12 cells were from Cell Resource Center of Shanghai Institute of Biological Sciences, Chinese Academy of Sciences. Cells were maintained in Dulbecco’s Modified Eagle Medium (HyClone) containing 10% fetal bovine serum (HyClone), 100 units/mL penicillin and 100 μg/mL streptomycin at 37°C in a humidified incubator under 95% air and 5% CO_2_.

### Construction of stably transfected cells

Human SIRT6 cDNA construct was cloned into pCDH-CMV-MCS-EF1-copGFP backbone. HEK293T cells were transfected with the pCDH constructs accompanied by pMD.2G/psPAX2 plasmids and mature lentivirus was obtained by ultracentrifugation. SH-SY5Y cells were transfected with the vector or SIRT6 lentivirus and stably transfected cells were selected by flow cytometry.

### Lactate dehydrogenase (LDH) assay

For intracellular LDH assay, cells were incubated with lysis buffer for 20 min. Cell lysates were mixed with reaction buffer containing 7.5 mmol/L sodium pyruvate and 1.5 mmol/L NADH at a ratio of 1:3, and the absorbance at 340 nm was measured over 30 s using a microplate reader (Synergy2, BioTek). For LDH release assay, cell culture medium was mixed with reaction buffer at a ratio of 1:1 and the absorbance at 340 nm was measured as above.

### CCK-8 cell viability assay

Cell viability was measured by using Cell Counting Kit-8 (Dojindo Laboratories, Kumamoto, Japan). In brief, cells were seeded in 96-well plates at the density of 1 × 10^4^/well. After treatment, CCK-8 solution was added to the medium at a dilution of 1:10 and cells were incubated at 37°C for 4 h. The absorbance at 450 nm was measured using a microplate reader (Synergy2, BioTek).

### Annexin V/7-AAD assay

The degrees of both apoptosis and necrosis were measured using ApoScreen Annexin V kit (Southern-Biotech, Birmingham, AL, USA). Briefly, cells were resuspended in pre-chilled binding buffer at concentrations of 5 × 10^6^ cells/mL. Annexin V solution was added into cell suspension at a dilution of 1:10. After incubation on ice for 15 min, 7-AAD solution was added into cell suspension at a dilution of 1:40. Further analysis was performed on a flow cytometer (BD FACSAriaII).

### Dihydroethidium (DHE) staining

Reactive oxygen species (ROS) levels were detected by DHE staining. In brief, after treatment cells were washed once with PBS, followed by incubation with 5 µmol/L DHE (Beyotime Institute of Biotechnology, Jiangsu Province, China) at 37°C for 20 min. Cells were then washed once with PBS again and the absorbance at 545 nm was obtained using a laser-scanning microscope (Leica TCS SP5 II, Germany).

### Assessment of autophagosome formation

Cells were seeded on coverslips in 24-well plates and transfected with EGFP-LC3 plasmid (0.5 µg for each well of a 24-well plate). Twenty-four hours after transfection, cells were exposed to H_2_O_2_ for 1 h and cultured for another 24 h. Cells were subsequently washed with PBS and fixed in 4% paraformaldehyde for 10 min. Finally slides were mounted with cover slips and epifluorescence images were taken using a confocal microscope (Leica, Solms, Germany). To quantify autophagosome formation, the number of EGFP-LC3 puncta per cell was counted in multiple fields.

### Real-time PCR

Total RNA was extracted from cells by RNAiso Plus (TaKaRa, Dalian, China) and reverse-transcribed to cDNA by PrimeScript RT reagent kit (TaKaRa). Quantitative real-time PCR was used to determine Sirt6 mRNA levels by SYBR Premix Ex Taq (TaKaRa) and the primers below: human SIRT6 (forward 5′-cccggatcaacggctctatc-3′ and reverse 5′-gccttcacccttttggggg-3′); human GAPDH (forward 5′-atggggaaggtgaaggtcg-3′ and reverse 5′-ggggtcattgatggcaacaata-3′).

### Western blot

Cells were lysed using RIPA buffer supplemented with protease inhibitors. 40 μg of total protein was used for Western blot analysis based on standard procedures. Protein signals were detected by ECL system. The primary antibodies were listed as follows: SIRT6 (1:1000) (Abcam, New Territories, HK); LC3 (1:2000), p62 (1:2000) (Sigma, MO, USA); AKT1 (1:1000), phos-AKT1 (1:1000) (Epitomics); ERK1/2 (1:1000), phos-ERK1/2 (1:2000), ATG5 (1:1000) (Cell Signaling Technology, Danvers, USA); beta-Tubulin (1:2000) (Abmart); Actin (1:500) (Santa Cruz Biotechnology, CA, USA).

### Plasmid or siRNA transfection

Cells were seeded at 40,000 cells per 24-well plate or 200,000 cells per 6-well plate. Cells were grown for 24 h and transfection was performed using Lipofectamine 2000 (Invitrogen) according to the manufacturer’s protocols. 4–6 h later, cells were replaced with fresh medium and cultured for 48 h to conduct further experiments. siRNA sequences were as follows: hAtg5 siRNA#1 (5′-GACCAUGCAAUGGUGGCUU-3′); hAtg5 siRNA#2 (5′-GCAGUGGCUGAGUGAACAU-3′); hAtg5 siRNA#3 (5′-GAUGCAAUUGAAGCUCAUU-3′); hSirt6 siRNA#1 (5′-GAAUGUGCCAAGUGUAAGATT-3′); hSirt6 siRNA #2 (5′-GGCUCUGCACCGUGGCUAATT-3′).

### Statistical analysis

Data were presented as mean ± SEM and analysed by ANOVA followed by Tukey’s post hoc test. *P* values less than 0.05 were considered statistically significant.

## Electronic supplementary material

Below is the link to the electronic supplementary material.

Supplementary material 1 (PDF 193 kb)
