# Erratum to: Autophagy induction by SIRT6 is involved in oxidative stress-induced neuronal damage

**DOI:** 10.1007/s13238-017-0370-1

**Published:** 2017-01-20

**Authors:** Jiaxiang Shao, Xiao Yang, Tengyuan Liu, Tingting Zhang, Qian Reuben Xie, Weiliang Xia

**Affiliations:** State Key Laboratory of Oncogenes and Related Genes, Renji-Med X Stem Cell Research Center, Ren Ji Hospital, School of Biomedical Engineering, Shanghai Jiao Tong University, Shanghai, 200030 China

## Erratum to: Protein Cell 2016, 7(4):281–290 DOI 10.1007/s13238-016-0257-6

The authors of this article would like to request the following replacement to the section ACKNOWLEGEMENTS:

We are grateful to Mrs. Jin Xu for her assistance in flow cytometry. This study is supported by the National Natural Science Foundation of China (Grant No. 31270032), the National Basic Research Program (973 Program) (No. 2013CB945604), and SJTU Interdisciplinary Research Grant (YG2012ZD05).

