# Supplementary material for: Autophagy induction by SIRT6 is involved in oxidative stress-induced neuronal damage
Source: Protein Cell. 2016 Mar 16;7(4):281–90. doi: 10.1007/s13238-016-0257-6 (PMC4818841; doi:10.1007/s13238-016-0257-6)
Supplement: Supplementary file 1 — Supplementary material 1 (PDF 193 kb) [file 13238_2016_257_MOESM1_ESM.pdf]

Supplementary Information

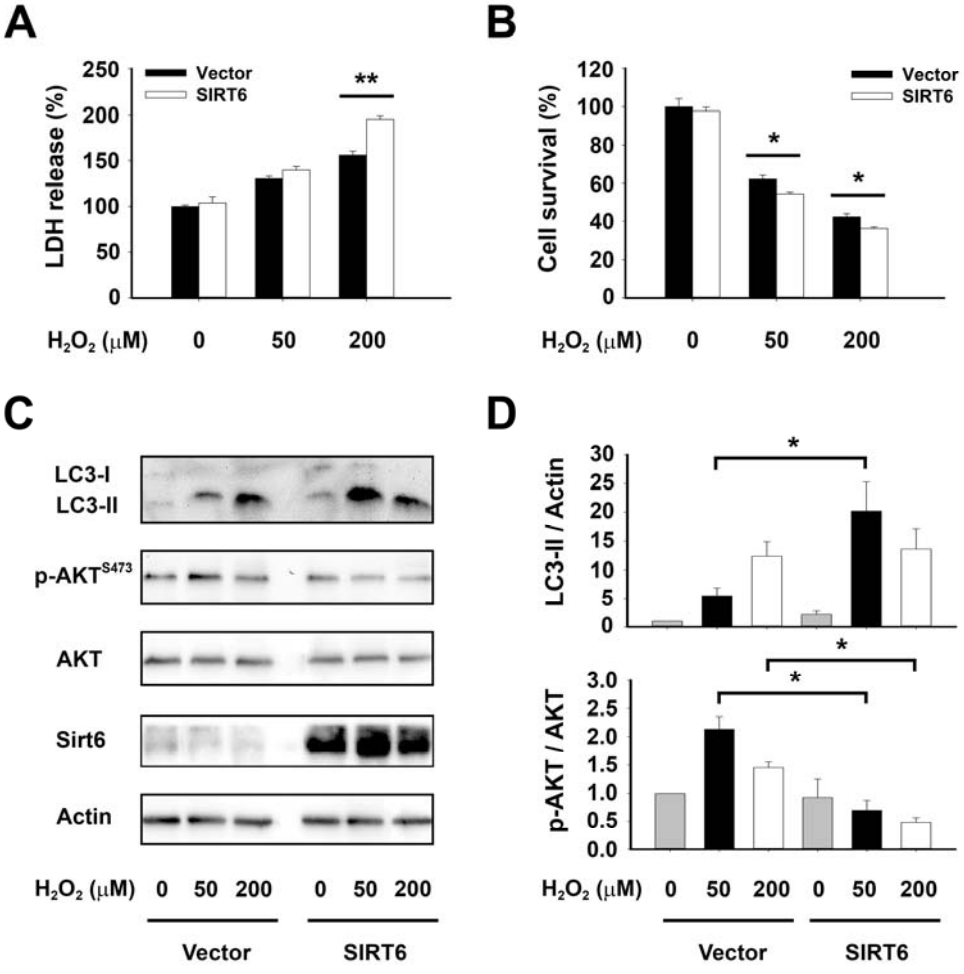

Fig S1. SIRT6 enhances H_2O_2-induced cell death through induction of autophagy in PC12 cells. (A and B) PC12 cells were exposed to H_2O_2 for 1 h and then cultured for 24 h after transfection with SIRT6 plasmid. Cell death was assessed by LDH release assay and cell viability was assessed by.CCK-8 assay. (C and D) PC12 cells were exposed to H_2O_2 for 1 h and then cultured for 24 h after transfection with SIRT6 plasmid. LC3 and phosphorylated AKT levels were analyzed by western blot and quantified. Bars represent the mean ± SEM from at least three independent experiments. *P<0.05, **P<0.01.

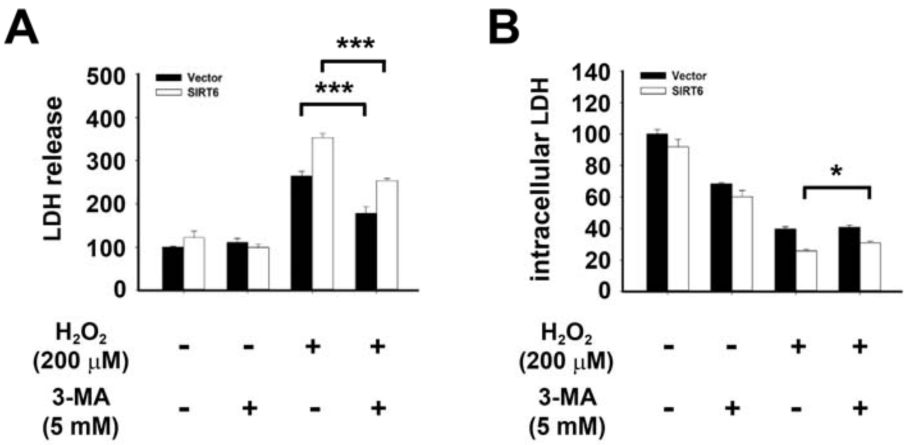

Fig S2. Inhibition of autophagy reduces H_2O_2-induced neuronal damage. (A and B) SH-SY5Y cells were pretreated with H_2O_2 for 1 h and then incubated with 3-MA for 24 h. Cell death and viability were assessed by LDH assay. Bars represent the mean ± SEM from at least three independent experiments. *P<0.05; ***P<0.001.

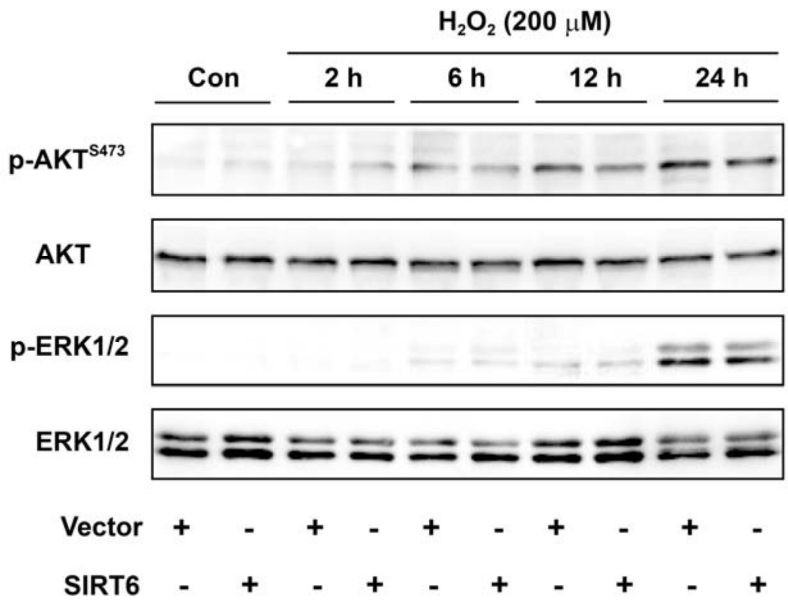

Fig S3. H_2O_2 treatment causes AKT and ERK MAPK activation. SH-SY5Y cells were pretreated with H_2O_2 for 1 h and then cultured for indicated time. Levels of phosphorylated AKT and ERK were analyzed by western blot.

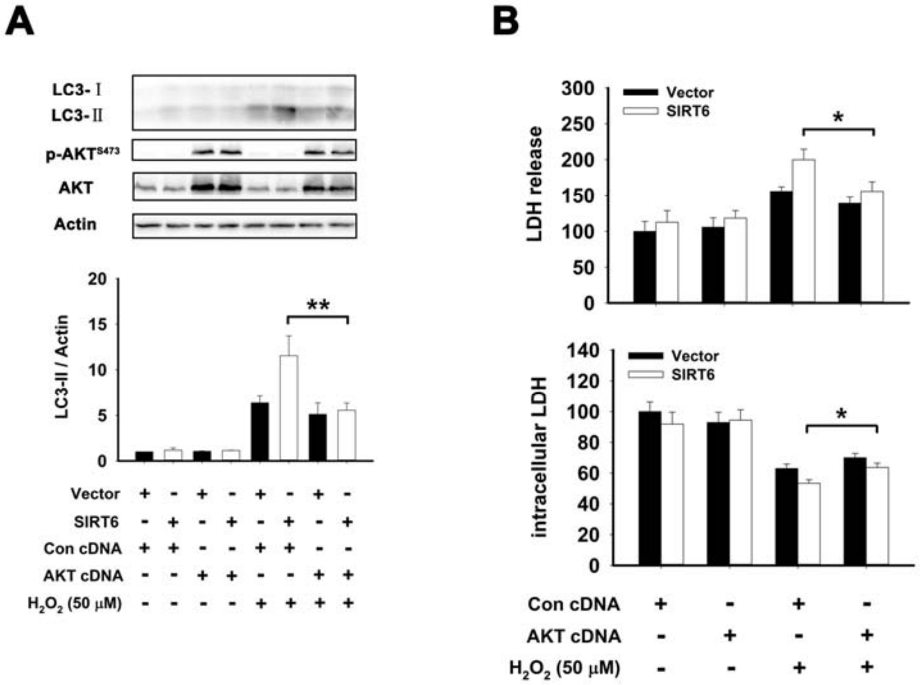

Fig S4. Activation of AKT signaling attenuates SIRT6-mediated autophagy and neuronal damage following lower concentration of H_2O_2 treatment. (A) SH-SY5Y cells were exposed to 50 μM H_2O_2 for 1 h and then cultured for 24 h after transfection with MYR-AKT plasmid. LC3 level was analyzed by western blot and quantified. (B) SH-SY5Y cells were exposed to 50 μM H_2O_2 for 1 h and then cultured for 24 h after transfection with MYR-AKT plasmid. Cell death and viability were assessed by LDH assay. Bars represent the mean ± SEM from at least three independent experiments. *P<0.05; **P<0.01.
